# Supplementary material for: A hierarchical atlas of the human cerebellum for functional precision mapping
Source: Nat Commun. 2024 Sep 27;15:8376. doi: 10.1038/s41467-024-52371-w (PMC11436828; doi:10.1038/s41467-024-52371-w)
Supplement: Supplementary file 1 — Supplementary tables and figures [file 41467_2024_52371_MOESM1_ESM.pdf]

SUPPLEMENTARY TABLES AND FIGURES

Supplementary Tables

Name	Subjects	Age	Conditions	min/subject	Field, Voxel size	Description
MDTB	24 (16 female)	23.8 ±2.6	62	320	3T, 3mm	Cognitive, motor, perceptual, social
Highres-MDTB	8 (3 female)	25.37±3.74	9	120	7T, 1.5mm	Cognitive, motor, perceptual, social
Nishimoto	6 (2 female)	22-33	103	162	3T, 2mm	Cognitive, motor, perceptual, social
IBC	12 (2 female)	34.5±4.9	208	822	3T, 1.5mm	Cognitive, motor, perceptual, social
WM	16 (8 female)	25±2	17	65	3T, 3mm	Motor and working memory task
Multi-demand	37 (23 female)	25.9±4.7	12	100	3T, 2mm	Executive Tasks
Somatotopic	8 (6 female)	22.4±2.6	6	96	3T, 1.8/2.4	Motor
HCP-Unrelated 100	100 (54 female)	29.1±3.7	none	60	3T, 2mm	Resting-state

Supplementary table 1. FMRI datasets used for the functional fusion. All datasets but the last are task-based. The last one refers to resting-state data from a subset of the HCP dataset.

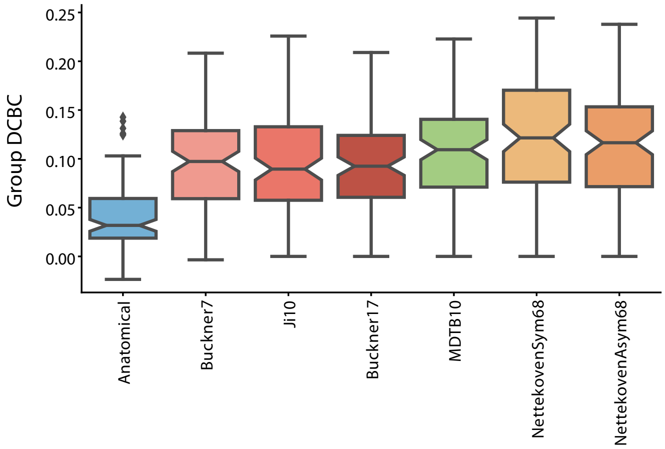

Figure S1. Fused atlas performance compared to existing atlases. DCBC evaluation of existing anatomical parcellation (Lobular: [5]), task-based parcellation (MDTB: [3], and resting-state parcellations (7 and 17 regions: [7]; 10 regions: [6]) averaged across all subjects (n=111 for each bar). The box is drawn from quartile 1 to quartile 3 with a horizontal line denoting the median. Whiskers represent 1.5 times interquartile range

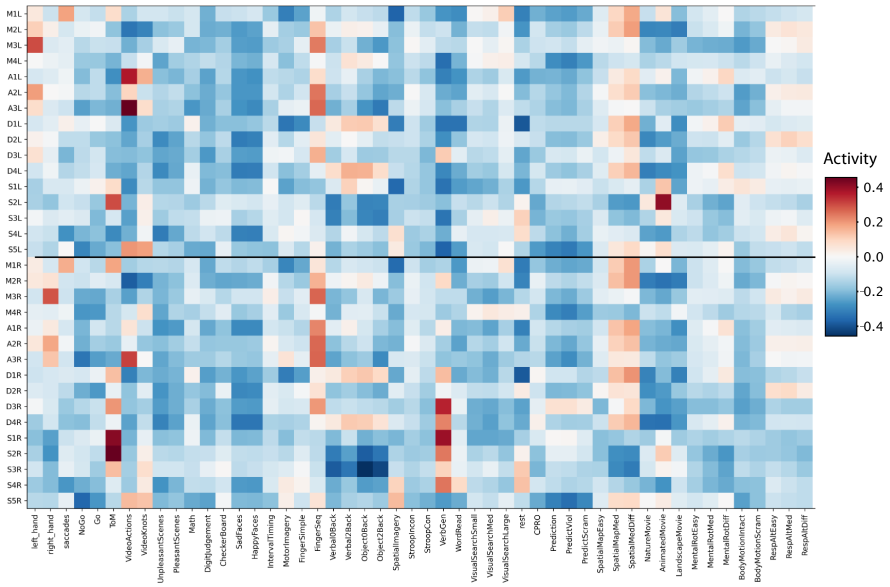

Figure S2. Functional profiles of regions in MDTB dataset. Average activity relative to the mean activity in all tasks in MDTB dataset corrected for motor features. Responses were estimated from subject-specific regions and averaged across subjects for visualization. To account for activation that can be explained by the motor aspects of each task, number of movements were used as covariates alongside regressors that coded for each condition separately. Movements were left hand presses, right hand presses and saccades per second.

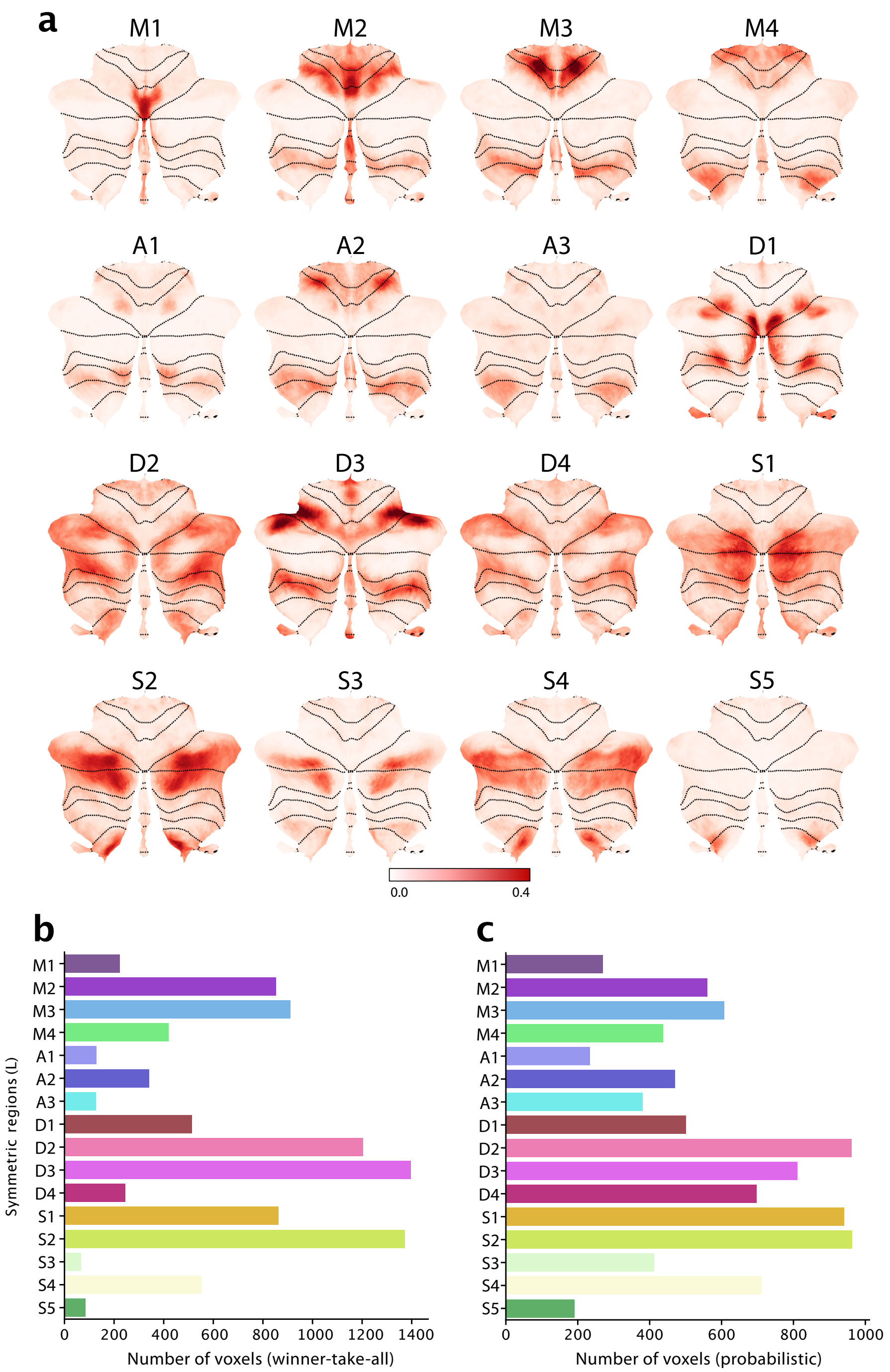

Figure S3. Probability maps and region size. **a**, Probability maps for each region displayed on the flat representation. **b**, Size estimate for each region in terms of the number of voxels ($2mm^3$) using winner-take-all assignment. **c**, Size estimate for each region in terms of the number of voxels ($2mm^3$) using probabilistic assignments.

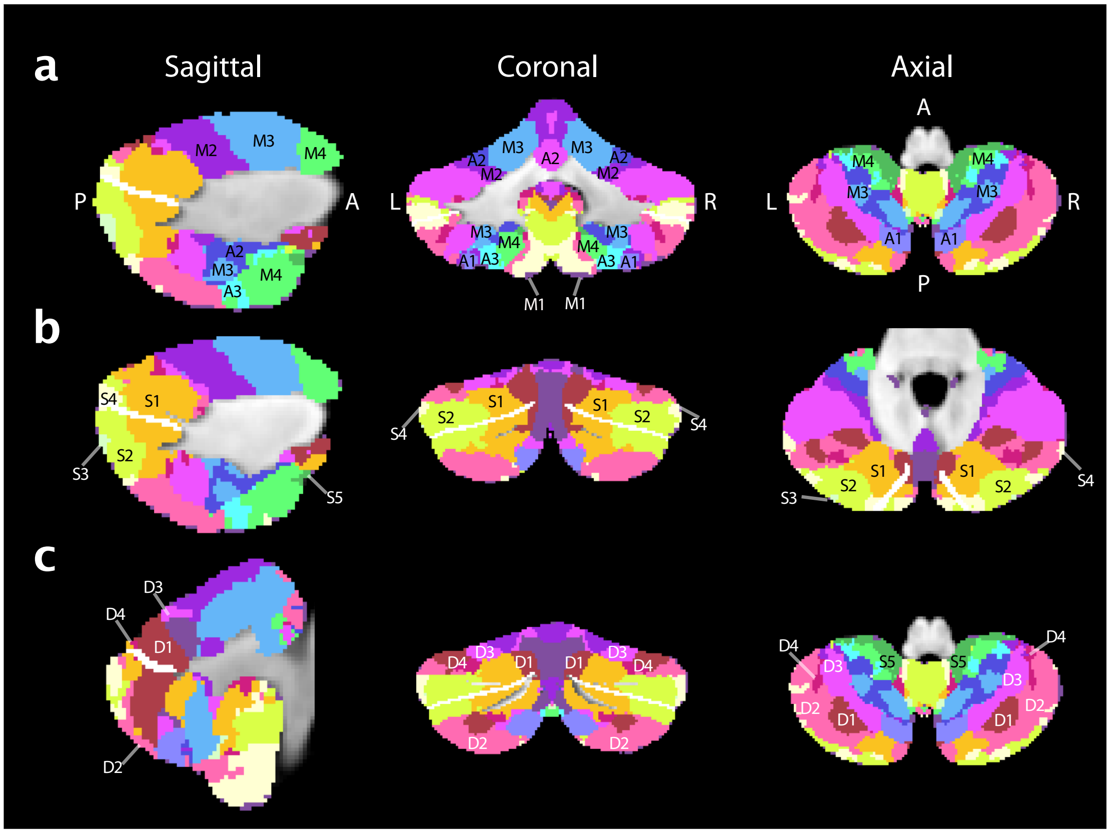

Figure S4. **Atlas in volumetric space**. Atlas shown at medium granularity (32 regions; 16 per hemisphere). **a**, Motor and action regions. **b**, Multi-demand regions. **c**, Social-linguistic-spatial regions. Horizontal fissure is marked in white.

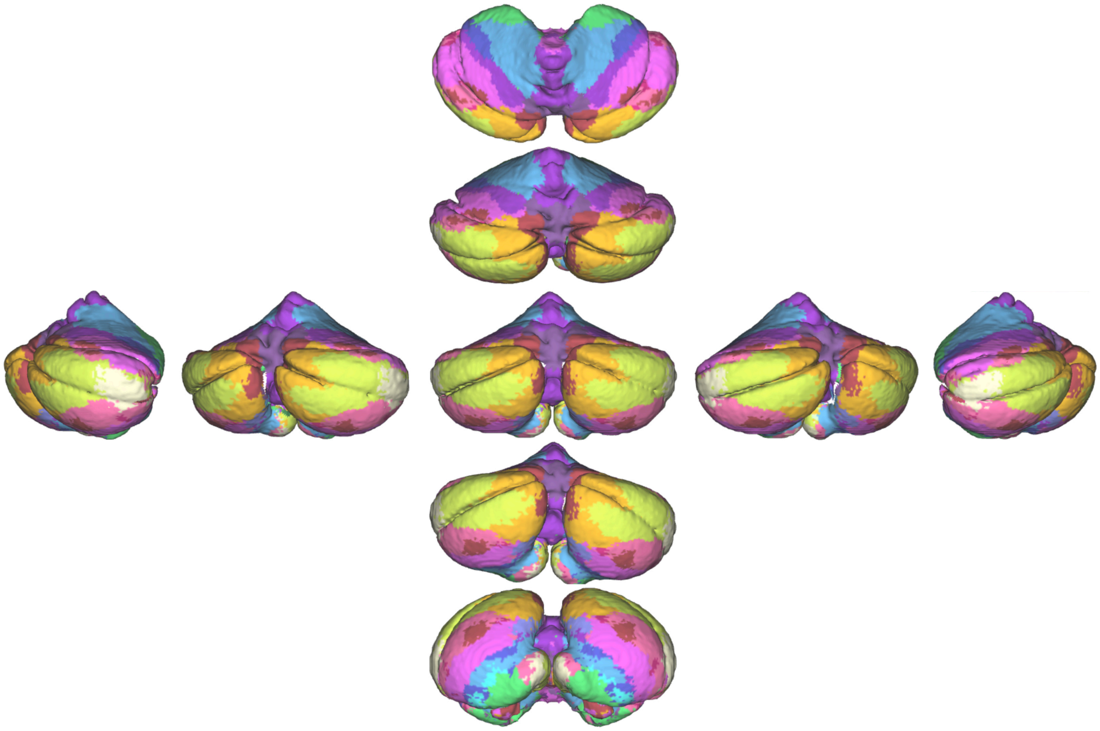

Figure S5. Atlas in 3D view. Atlas shown at medium granularity (32 regions; 16 per hemisphere) projected onto the pial surface. The central view is showing the posterior side of the cerebellum. The vertically arranged views show the superior side of the cerebellum at the top and the inferior side at the bottom. The horizontally arranged views show the left and right side of the cerebellum.

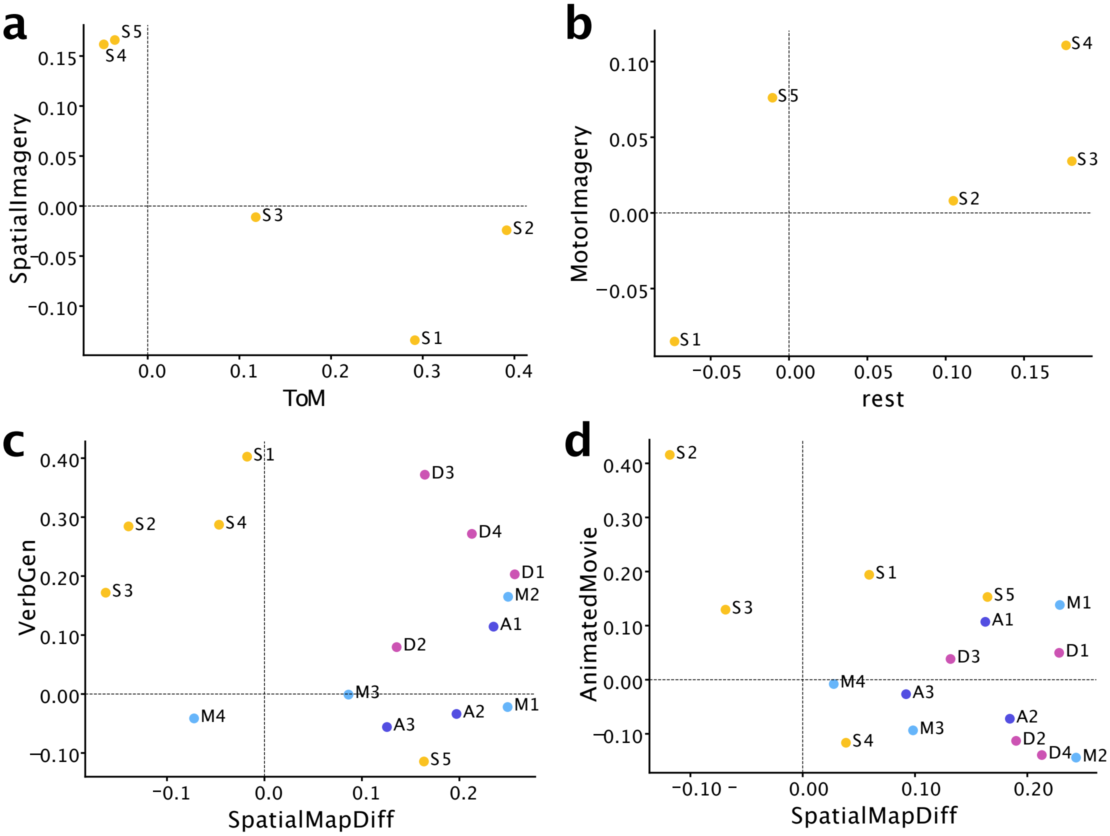

Figure S6. **Regional differences in functional responses for selected tasks**. **a-b**, Spatial imagery, theory-of-mind, motor imagery and rest separate social-linguistic-spatial (S1-5) regions. **c-d**, Verb generation, spatial map, and animated movie tasks seperate social-linguistic-spatial regions from other domains. For **c**, only the right regions are shown and for **d** only the left regions are shown. For the other panels the responses are shown averaged across hemispheres.

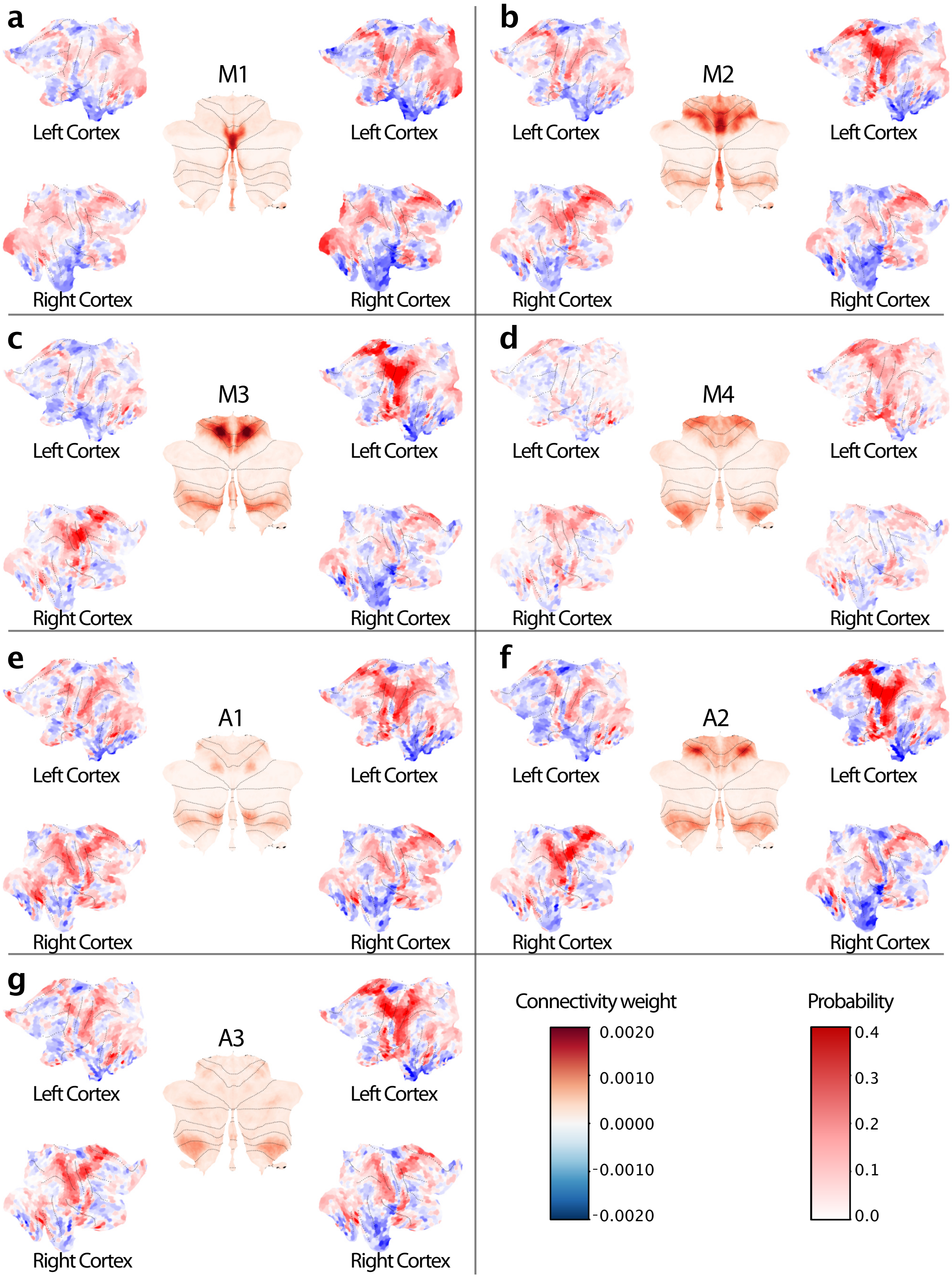

Figure S7. Cortico-cerebellar connectivity weights and probability maps. Parcel probability maps for motor (**a-d**) and action (**e-g**) regions are shown in the middle of each figure inset, surrounded by the cortical input weights for the left and right cerebellar parcel. Weights for the left cerebellar parcel are shown to the left of the probability map and for the right cerebellar parcel to the right of each probability map on the cortical flatmap. Motor regions include oculomotor vermis M1 (**a**), tongue and vermal region M2 (**b**), hand M3 (**c**) and lower body M4 (**d**) region. Action regions include spatial simulation regions A1 (**e**), classical action observation A2 (**f**) and motor imagery region A3 (**g**).

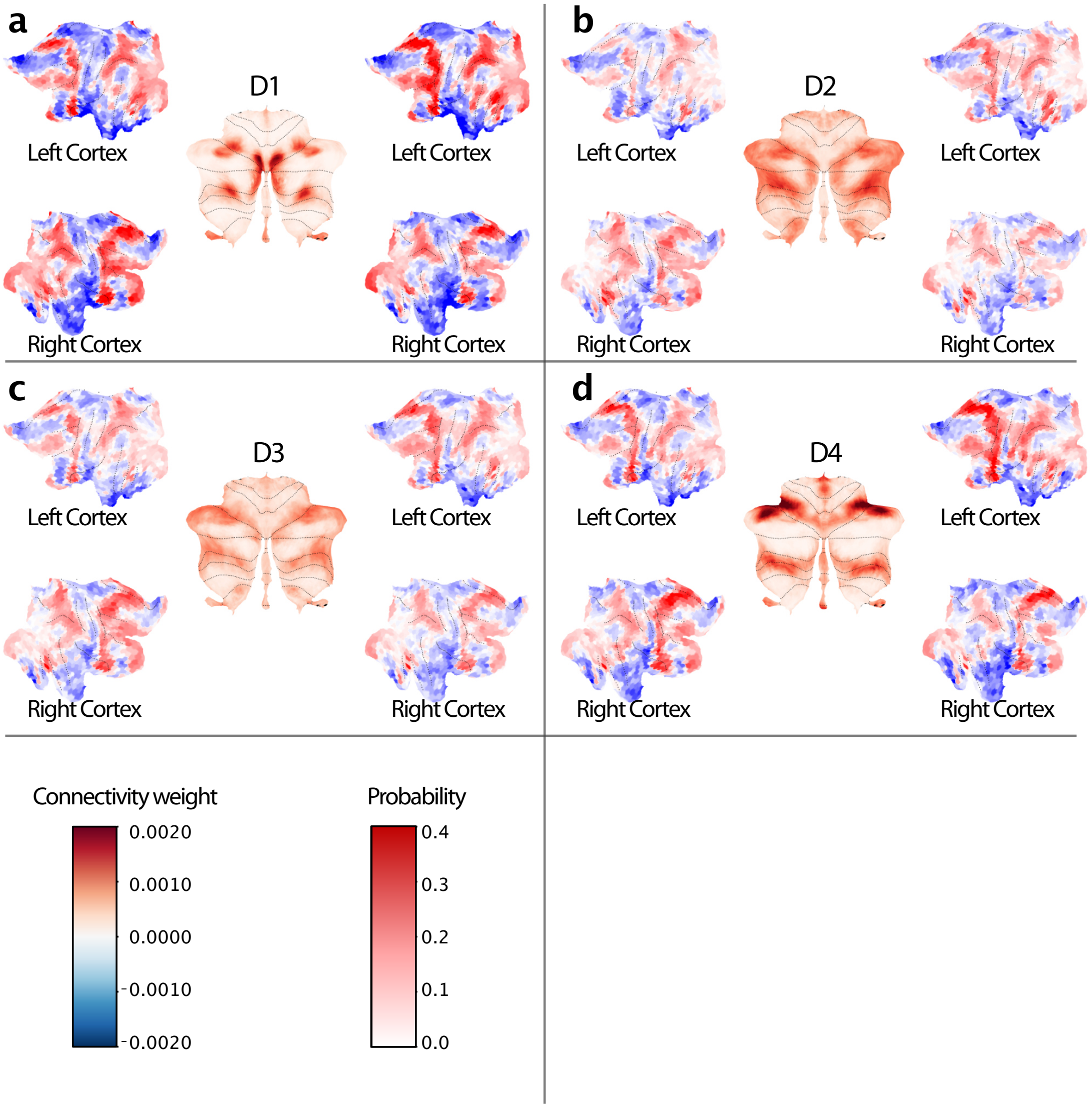

Figure S8. Cortico-cerebellar connectivity weights and probability maps for demand. a-d, Parcel probability maps for multiple demand regions are shown in the middle of each figure inset, surrounded by the cortical input weights for the left and right cerebellar parcel. Weights for the left cerebellar parcel are shown to the left of the probability map and for the right cerebellar parcel to the right of each probability map on the cortical flatmap. Demand regions include spatial working memory region (**a**), recall regions (**b**, difficulty-related (**c**) and n-back region (**d**) region.

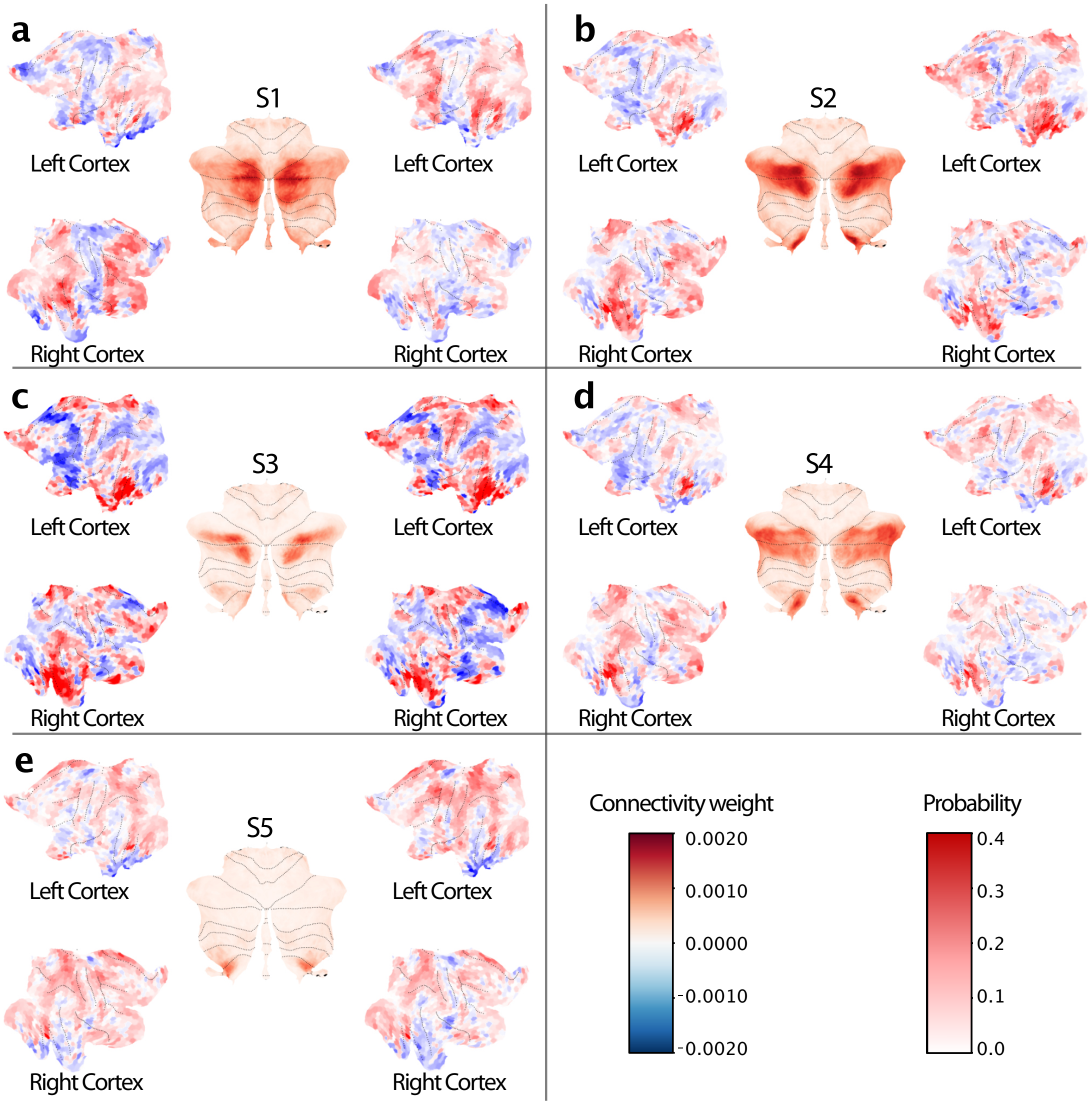

Figure S9. Cortico-cerebellar connectivity weights and probability maps for social-linguistic-spatial regions. a-e, Parcel probability maps for social-linguistic-spatial regions are shown in the middle of each figure inset, surrounded by the cortical input weights for the left and right cerebellar parcel. Weights for the left cerebellar parcel are shown to the left of the probability map and for the right cerebellar parcel to the right of each probability map on the cortical flatmap. Social-linguistic-spatial regions include linguistic region S1 (**a**), social region S2 (**b**), rest region S3 (**c**), self-projection region S4 (**d**) and scene construction region S5 (**e**).

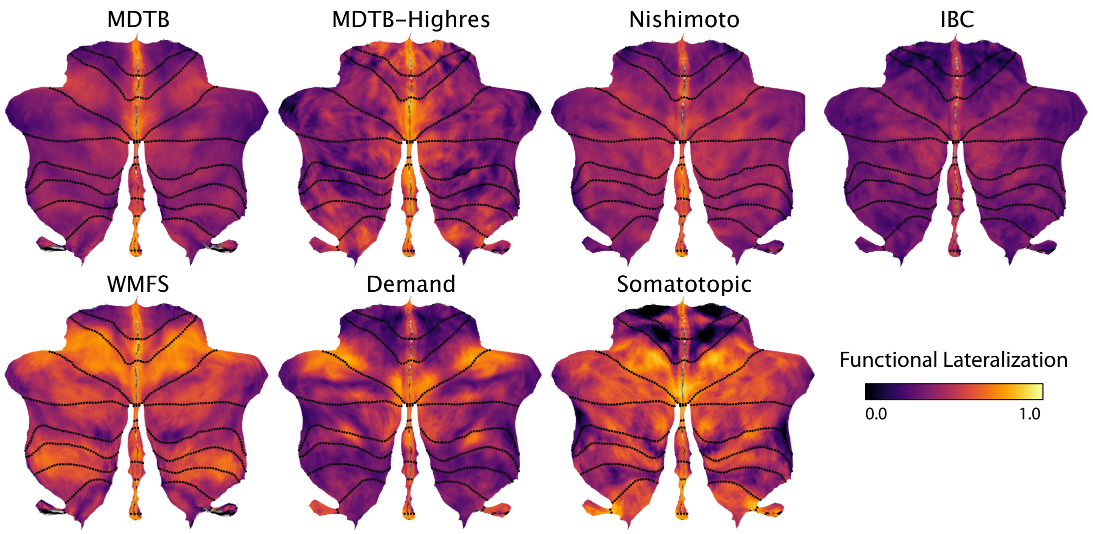

Figure S10. Individual functional lateralization for each dataset. Functional lateralization calculated as the correlations of the functional responses of anatomically corresponding voxel of the left and right hemisphere. Functional lateralization was averaged across subjects within each dataset.

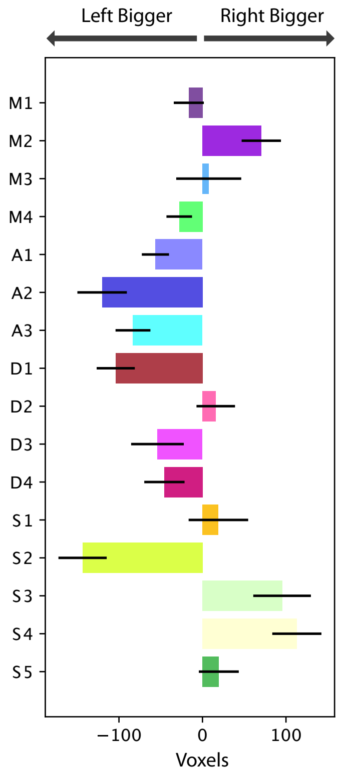

Figure S11. Size difference between left and right region pairs of the asymmetric atlas. Regions were estimated in individual subjects using the asymmetric atlas version. The size difference was calculated as number of voxels ($2mm^3$) in right parcel minus number of voxels in left parcel for each individual. Bars show average size difference across individuals and error bars indicate standard error of the mean across subjects (n=111 for each bar).
