# Supplementary material for: A hierarchical atlas of the human cerebellum for functional precision mapping
Source: Nat Commun. 2024 Sep 27;15:8376. doi: 10.1038/s41467-024-52371-w (PMC11436828; doi:10.1038/s41467-024-52371-w)
Supplement: Supplementary file 3 — Reporting Summary [file 41467_2024_52371_MOESM3_ESM.pdf]

nature portfolio

Corresponding author(s): Nettekoven, Caroline; Diedrichsen, Joern

Last updated by author(s): Mar 6, 2024

Reporting Summary

Nature Portfolio wishes to improve the reproducibility of the work that we publish. This form provides structure for consistency and transparency in reporting. For further information on Nature Portfolio policies, see our Editorial Policies and the Editorial Policy Checklist.

Statistics

For all statistical analyses, confirm that the following items are present in the figure legend, table legend, main text, or Methods section.

n/a	Confirmed	
☐	☒	The exact sample size (*n*) for each experimental group/condition, given as a discrete number and unit of measurement
☐	☒	A statement on whether measurements were taken from distinct samples or whether the same sample was measured repeatedly
☐	☒	The statistical test(s) used AND whether they are one- or two-sided<br>*Only common tests should be described solely by name; describe more complex techniques in the Methods section.*
☐	☒	A description of all covariates tested
☐	☒	A description of any assumptions or corrections, such as tests of normality and adjustment for multiple comparisons
☐	☒	A full description of the statistical parameters including central tendency (e.g. means) or other basic estimates (e.g. regression coefficient) AND variation (e.g. standard deviation) or associated estimates of uncertainty (e.g. confidence intervals)
☐	☒	For null hypothesis testing, the test statistic (e.g. *F*, *t*, *r*) with confidence intervals, effect sizes, degrees of freedom and *P* value noted<br>*Give P values as exact values whenever suitable.*
☐	☒	For Bayesian analysis, information on the choice of priors and Markov chain Monte Carlo settings
☐	☒	For hierarchical and complex designs, identification of the appropriate level for tests and full reporting of outcomes
☐	☒	Estimates of effect sizes (e.g. Cohen's *d*, Pearson's *r*), indicating how they were calculated

Our web collection on statistics for biologists contains articles on many of the points above.

Software and code

Policy information about availability of computer code

Data collection

1. MDTB dataset: The 26 tasks were presented and behavioral data collected using custom-written code in MATLAB R2015b. The low-level functionality from psychtoolbox (3.0.13) was used for display and execution of the tasks tested in both the behavioural and fMRI sessions. The Eyelink toolbox (MATLAB toolbox) was used to collect eye movement data.
2. Highres-MDTB dataset: The 9 tasks were presented and behavioral data collected using custom-written code in PsychoPy. PsychoPy was used for display and execution of tasks tested both in behavioral and fMRI sessions.
3. Nishimoto dataset: Siemens MAGNETOM TrioTim (Siemens, syngo MR B17) and Presentation (Neurobehavioral Systems, ver. 18.0)
4. Individual Brain Charting (IBC) dataset: Metadata, concerning the stimuli presented during the BOLD fMRI runs, were made available publicly at https://github.com/hbp-brain-charting/public_protocols. They include: (1) the task-stimuli protocols; (2) demo presentations of the tasks as video annotations; (3) instructions to the participants; and (4) scripts to extract paradigm descriptors from log files for the GLM estimation.
5. WMFS dataset: We used tools from SPM12 and custom written code in MATLAB 2018b to process the functional and anatomical data process the functional and anatomical data.
6. Multi-Demand dataset: MRI CCF acquisition protocols for HCP Young Adult cohort were used (package date 2016.07.14; https://protocols.humanconnectome.org/CCF/).
7. Somatotopic mapping dataset: Siemens Magnetom Prisma-fit MRI scanner and a 64-channel phased-array head-neck coil (Siemens Healthcare, Erlangen, Germany). The Eyelink 1000 Core Plus with Long-Range Mount (SR Research, Ottawa, Ontario,Canada), and alertness was scored during each functional run.
8. HCP Unrelated 100 dataset: The HCP resting-state data is from the publicly available Human Connectome Dataset at https://www.humanconnectome.org/study/hcp-young-adult/data-releases

Data analysis

SPM12 and SUIT (3.3) toolboxes were used to analyze the data. The code for building the atlas and generating the results and figures in this paper is publicly available as the GitHub repository https://github.com/DiedrichsenLab/ProbabilisticParcellation. The code for the hierarchical Bayesian parcellation framework is available at https://github.com/DiedrichsenLab/HierarchBayesParcel. The organization, file system, and code for managing the diverse set of datasets is available at https://github.com/DiedrichsenLab/Functional_Fusion.

For manuscripts utilizing custom algorithms or software that are central to the research but not yet described in published literature, software must be made available to editors and reviewers. We strongly encourage code deposition in a community repository (e.g. GitHub). See the Nature Portfolio guidelines for submitting code & software for further information.

Data

Policy information about availability of data

All manuscripts must include a data availability statement. This statement should provide the following information, where applicable:

- Accession codes, unique identifiers, or web links for publicly available datasets
- A description of any restrictions on data availability
- For clinical datasets or third party data, please ensure that the statement adheres to our policy

The raw data for the fMRI studies used in this project are publicly available at OpenNeuro.org (https://openneuro.org/). Specifically, MDTB (https://openneuro.org/datasets/ds002105/versions/1.1.0), Nishimoto (https://openneuro.org/datasets/ds002306/versions/1.0.3), IBC (https://openneuro.org/datasets/ds002685/versions/1.3.1), HCP unrelated 100 (https://www.humanconnectome.org/study/hcp-young-adult/data-releases). Three datasets can be found in their original papers: WMFS (https://doi.org/10.1101/2023.01.25.525395), Multi-Demand dataset (https://doi.org/10.1101/2022.12.01.518720), and Somatotopic mapping dataset (https://doi.org/10.1152/jn.00165.2022). The High-res MDTB dataset has not yet been published.

Research involving human participants, their data, or biological material

Policy information about studies with human participants or human data. See also policy information about sex, gender (identity/presentation), and sexual orientation and race, ethnicity and racism.

Reporting on sex and gender

1. MDTB dataset (16 females and 8 males)
2. Highres-MDTB dataset (3 females and 5 males)
3. Nishimoto dataset (2 females and 4 males)
4. Individual Brain Charting (IBC) dataset (2 females and 10 males)
5. WMFS dataset (8 females and 8 males)
6. Multi-Demand dataset (23 females and 14 males)
7. Somatotopic mapping dataset (6 females and 2 males)
8. HCP Unrelated 100 dataset (54 females and 46 males)

Reporting on race, ethnicity, or other socially relevant groupings

1. MDTB dataset: 24 healthy right-handed adults (mean age=23.8 years old, SD=2.6) with no self-reported history of neurological or psychiatric illness.
2. Highres-MDTB dataset: 8 healthy right-handed adults (mean age= 25.37 years old, SD=3.74) with no self-reported history of neurological or psychiatric illness.
3. Nishimoto dataset: Research participants were Osaka University students and adjuncts (4 males and 2 females, aged 22-33). All participants were healthy, had normal vision and hearing ability, and passed institutional pre-screening procedure for MRI experiments (e.g., no metal implants, not pregnant, etc.)
4. Individual Brain Charting (IBC) dataset: 12 healthy adults (mean age=34.5 yrs, sd=4.9) with 11 right-handed.
5. WMFS dataset: 16 healthy participants (mean age = 25, std age = 2) with right-handed.
6. Multi-Demand dataset: 37 subjects (mean age=25.9, sd=4.7). All subjects had normal or corrected vision (using MRI compatible glasses)
7. Somatotopic mapping dataset: 8 healthy adults (aged 19–25, means = 22.4 yr, SD =2.6, 7 right-handed). All participants were screened to exclude a history of neurological and psychiatric illness or ongoing use of psychoactive medications.
8. HCP Unrelated 100 dataset: 100 healthy subjects with (mean age=29.1 yrs, sd=3.7 yrs)

Population characteristics

1. MDTB dataset: 24 healthy right-handed adults (mean age=23.8 years old, SD=2.6) with no self-reported history of neurological or psychiatric illness.
2. Highres-MDTB dataset: 8 healthy right-handed adults (mean age= 25.37 years old, SD=3.74) with no self-reported history of neurological or psychiatric illness.
3. Nishimoto dataset: Research participants were Osaka University students and adjuncts (4 males and 2 females, aged 22-33). All participants were healthy, had normal vision and hearing ability, and passed institutional pre-screening procedure for MRI experiments (e.g., no metal implants, not pregnant, etc.)
4. Individual Brain Charting (IBC) dataset: 12 healthy adults (mean age=34.5 yrs, sd=4.9) with 11 right-handed.
5. WMFS dataset: 16 healthy participants (mean age = 25, std age = 2) with right-handed.
6. Multi-Demand dataset: 37 subjects (mean age=25.9, sd=4.7). All subjects had normal or corrected vision (using MRI compatible glasses)
7. Somatotopic mapping dataset: 8 healthy adults (aged 19–25, means = 22.4 yr, SD =2.6, 7 right-handed). All participants were screened to exclude a history of neurological and psychiatric illness or ongoing use of psychoactive medications.
8. HCP Unrelated 100 dataset: 100 healthy subjects with (mean age=29.1 yrs, sd=3.7 yrs)

Recruitment

1. MDTB dataset: Undergraduate and graduate students were recruited (via posters) from the larger student body at Western University. Thus, our sample was biased towards relatively high-functioning, healthy and young individuals. While we don't expect cerebellar organization to be dramatically different in this group, caution needs to be exercised when generalizing the results to the general population.

2. Highres-MDTB dataset: Graduate students were recruited (via posters and word-of-mouth) from the larger student body at Western University. Thus, our sample was biased towards relatively high-functioning, healthy and young individuals. While we do not expect cerebellar organization to be dramatically different in this group, caution needs to be exercised when generalizing the results to the general population.
3. Nishimoto dataset: Participants were recruited from a local participant pool under the following selection criteria: (1) a participant can join at least three fMRI sessions and (2) a participant is healthy and with normal vision and hearing.
4. Individual Brain Charting (IBC) dataset:The twelve participants were recruited by poster advertisements in the local area. Exclusion criteria were: (i) IQo80 or IQ>130; (ii) the use of drugs prior to the first exam; (iii) participation in other research protocol involving drugs; (iv) psychiatric and neurologic disorders requiring medication with potential impact on general cognitive abilities; (v) hearing problems; and (vi) any standard MRI counter-indications
5. WMFS dataset: Undergraduate/graduate students were recruited (via posters) from the larger student body at Western University. Thus, our sample was biased towards relatively high-functioning, healthy and young individuals.
6. Multi-Demand dataset: N/A
7. Somatotopic mapping dataset: The eight healthy adults were recruited from the Boston area.
8. HCP Unrelated 100 dataset: Our primary participant pool comes from healthy individuals born in Missouri to families that include twins, based on data from the Missouri Department of Health and Senior Services Bureau of Vital Records. Additional recruiting efforts are used to insure that participants broadly reflect the ethnic and racial composition of the U.S. population as represented in the 2000 decennial census.

Ethics oversight

1. MDTB dataset: The Ethics committee at Western University approved all experimental protocols (Protocol number: 107293).
2. Highres-MDTB dataset: The Ethics committee at Western University approved all experimental protocols (Protocol number: 107293).
3. Nishimoto dataset: National Institute of Information and Communications Technology
4. Individual Brain Charting (IBC)dataset: The experimental procedures were approved by a regional ethical committee for medical protocols in Île-de-France and a committee to ensure compliance with data-protection rules. All participants were undertaken with the informed written consent of each participant according to the Helsinki declaration and the French public health regulation.
5. WMFS dataset: The Ethics committee at Western University approved all experimental protocols (Protocol number: 107293).
6. Multi-Demand dataset: Informed consent was obtained from each subject and the study was approved by the Cambridge Psychology Research Ethics Committee.
7. Somatotopic mapping dataset: Paid participants provided written informed consent through a protocol approved by the Institutional Review Board of Harvard University
8. HCP Unrelated 100 dataset: NIH Neuroscience Blueprint Institutes and Centers. All subjects gave written informed consent to the Human Connectome Project consortium

Note that full information on the approval of the study protocol must also be provided in the manuscript.

Field-specific reporting

Please select the one below that is the best fit for your research. If you are not sure, read the appropriate sections before making your selection.

☒ Life sciences ☐ Behavioural & social sciences ☐ Ecological, evolutionary & environmental sciences

For a reference copy of the document with all sections, see nature.com/documents/nr-reporting-summary-flat.pdf

Life sciences study design

All studies must disclose on these points even when the disclosure is negative.

Sample size

1. MDTB dataset: N=24 subjects, 5.5 hrs fMRI data per subject, 62 unique task conditions
2. Highres-MDTB dataset: N=8 subjects, 2 hrs fMRI data per subject, 9 unique task conditions
3. Nishimoto dataset: N=6 subjects, 162 minutes fMRI data per subject, 103 unique task conditions
4. Individual Brain Charting (IBC) dataset: N=12 subjects, 822 minutes fMRI data per subject, 208 unique task conditions
5. WMFS dataset: N=16 subjects, 65 minutes fMRI data per subject, 17 unique task conditions
6. Multi-Demand dataset: N=37 subjects, 100 minutes fMRI data per subject, 12 unique task conditions
7. Somatotopic mapping dataset: N=8 subjects, 96 minutes fMRI data per subject, 6 unique task conditions
8. HCP Unrelated 100 dataset: N=100 subjects, 1 hr resting-state fMRI data per subject

Data exclusions

1. MDTB dataset: Originally recruited 31 participants. N=5 participants could not return to complete the second task set as they had moved. These data were not included in the final analyses. Two additional participants were excluded from the analyses as they failed to complete all 32 scanning runs for technical reasons. Eye-tracking data from two participants in set A and three participants in set B were not obtained due to technical problems.
2. Highres-MDTB dataset: Originally recruited 12 participants. N=4 participants either were excluded due either to data quality issues or not returning to complete the second scanning session.
3. Nishimoto dataset: Some data were excluded (and re-measured) when we detected the following technical issues during experiments: the earphone was not properly attached.
4. Individual Brain Charting (IBC) dataset: All tasks selected from this dataset were performed by 12 participants. We used all tasks reported in Pinho et al. 2018 plus all tasks reported in Pinho et al. 2020 but the Self and the Bang tasks. Additionally, we also used the following tasks: Wedge, Ring, LEC1, LEC2, Audi, Visu, Moto, MVEB, MVIS, MCSE, MathLanguage and SpatialNavigation. They are described in the IBC

	documentation: https://individual-brain-charting.github.io/docs/tasks.html<br>5. WMFS dataset: A total of 21 participants started the experiment. Of these, 4 participants were not scanned because of their poor performance during the behavioral training session. The remaining 17 participants performed the tasks inside the scanner and data for one participant was excluded due to an incidental finding.<br>6. Multi-Demand dataset: Originally fifty subjects were scanned over two sessions; thirteen subjects were excluded either due to incomplete data (n=5), excessive head movement during scanning (n=4; movement more than double the fMRI voxel size), technical problems during scanning (n=2; MRI scanner crashing) or during analysis (n=2; excessive field in homogeneities due to unreported teeth implants that affected structural scans).<br>7. Somatotopic mapping dataset: One motor run was excluded for S3,two motor runs for S4, one motor and two fixation runs for S6, and one fixation run for S8 due to motion. Runs were excluded based on BOLD data quality before examination of task response patterns to avoid bias.<br>8. HCP Unrelated 100 dataset: This is the subset of subjects provided by HCP S900 release ensures that they are not family relatives. It means the participants with family-structure co-variables were excluded.
Replication	1. MDTB dataset: The data includes a direct replication, as there are two identical sessions for each task set. There was good reliability of activation patterns across sessions (within a task set). Models were tested using prediction accuracy for a complete separate task set.<br>2. Highres-MDTB dataset: The data include a direct replication, as there are two identical sessions for the task set. There was good reliability of activation patterns across sessions.<br>3. Nishimoto dataset: The experiment consisted of 18 runs, with 12 training runs and 6 test runs. In the test runs, 103 tasks were presented four times in the same order across all six runs (but with different instances for each repetition).<br>4. Individual Brain Charting (IBC) dataset: Two different acquisitions for the same task were always performed using two opposite phase-encoding directions: one from Posterior to Anterior (PA) and the other from Anterior to Posterior (AP). The main purpose was to ensure within-subject replication of the same tasks, while mitigating potential limitations concerning the distortion-correction procedure.<br>5. WMFS dataset: the participant performed 5 imaging blocks of the finger tapping task, alternating with 5 blocks of the working memory task. Each block of the alternating finger tapping task lasted for just over 5 minutes, during which 260 volumes were collected. Conditions were fully randomized, each repeating 5 times within a block. Four 12-second periods of rest were interleaved randomly between trials.<br>6. Multi-Demand dataset: Both rest and task EPI runs were acquired in pairs of reversed phase-encoding directions (AP/PA).<br>7. Somatotopic mapping dataset: Data from the initial four participants were fully analyzed and graphed (Discovery sample,S1–S4) before any analysis was attempted on the second, independent group of participants (Replication sample,S5–S8)<br>8. HCP Unrelated 100 dataset: There are total two imaging sessions with each of them has two runs of 15 minutes resting-state scans, which allowing data averaging or for increased success in obtaining usable data.
Randomization	1. MDTB dataset: The sequence of task was randomized across imaging runs. All of the participants performed the same sequence of tasks (and the same number / order of runs) to enable analysis on the timeseries across participants.<br>2. Highres-MDTB dataset: The sequence of task was randomized across imaging runs. All of the participants performed the same sequence of tasks (and the same number / order of runs) to enable analysis on the timeseries across participants.<br>3. Nishimoto dataset: n/a (Participants were not allocated into experimental groups.)<br>4. Individual Brain Charting (IBC) dataset: All trials within each task were pseudo-randomized in order to avoid the extensively consecutive repetition of trials containing conditions of the same kind.<br>5. WMFS dataset: Each trial was randomly selected from one of five conditions.<br>6. Multi-Demand dataset: The same subjects performed all three tasks within the same session and within the same runs, enabling analysis on the time series across participants.<br>7. Somatotopic mapping dataset: n/a. All of the participants performed the same sequence of tasks<br>8. HCP Unrelated 100 dataset: n/a, each participant was scanned of 4 runs of 15 minutes of resting-state data.
Blinding	Blinding was not applicable. Participants in all datasets were not sorted into control and / experimental groups.

Reporting for specific materials, systems and methods

We require information from authors about some types of materials, experimental systems and methods used in many studies. Here, indicate whether each material, system or method listed is relevant to your study. If you are not sure if a list item applies to your research, read the appropriate section before selecting a response.

Materials & experimental systems

n/a	Involved in the study	
☒	☐	Antibodies
☒	☐	Eukaryotic cell lines
☒	☐	Palaeontology and archaeology
☒	☐	Animals and other organisms
☒	☐	Clinical data
☒	☐	Dual use research of concern
☒	☐	Plants

Methods

n/a	Involved in the study	
☒	☐	ChIP-seq
☒	☐	Flow cytometry
☐	☒	MRI-based neuroimaging

Plants

Seed stocks	*Report on the source of all seed stocks or other plant material used. If applicable, state the seed stock centre and catalogue number. If plant specimens were collected from the field, describe the collection location, date and sampling procedures.*
Novel plant genotypes	*Describe the methods by which all novel plant genotypes were produced. This includes those generated by transgenic approaches, gene editing, chemical/radiation-based mutagenesis and hybridization. For transgenic lines, describe the transformation method, the number of independent lines analyzed and the generation upon which experiments were performed. For gene-edited lines, describe the editor used, the endogenous sequence targeted for editing, the targeting guide RNA sequence (if applicable) and how the editor was applied.*
Authentication	*Describe any authentication procedures for each seed stock used or novel genotype generated. Describe any experiments used to assess the effect of a mutation and, where applicable, how potential secondary effects (e.g. second site T-DNA insertions, mosiacism, off-target gene editing) were examined.*

Magnetic resonance imaging

Experimental design

Design type

The first 7 datasets are task-based, HCP dataset is resting-state

Design specifications

1. MDTB dataset: Two task sets. 2 fMRI scanning sessions per task set. 8 functional imaging runs per session (10-min each). 17 tasks per imaging run (35 s each).
2. High-res MDTB dataset: One task set. 2 fMRI scanning sessions. 8 functional imaging runs per session (6-min each). 9 tasks per imaging run (35s each).
3. Nishimoto dataset: The main experiment was conducted in 3 separate fMRI sessions. The total of 18 runs were acquired across the three sessions. Of these, 12 runs were used to train voxel-wise models, and 6 runs were used to test the modeling accuracy. A single run consisted of 556 seconds. Stimuli used in the training and test runs were different.
4. Individual Brain Charting (IBC) dataset: 15 task sets.
5. WMFS dataset: 5 imaging blocks of the finger tapping task, alternating with 5 blocks of the working memory task. Each block of the alternating finger tapping task lasted for just over 5 minutes, during which 260 volumes were collected. Repeating 5 times within a block. Four 12-second periods of rest were interleaved randomly between trials
6. Multi-Demand dataset: 3 tasks were performed in the same scanning session: n-back, switch and stop signal. All three tasks were visual. 2 other tasks were performed in second session: an auditory version of the n-back task and a fifth task not relevant for this study. Each subject performed 4 runs in a session. Each run consisted of 36 blocks: 8 n-back, 8 switch, 8 stop and 12 fixation blocks. Each task consisted of 4 easy and 4 hard blocks. Each task block (30 s) started with a cue (4 s) followed by 12 trials (24 s, 2 s each) and ended with a blank screen (2 s) as an inter-block interval.
7. Somatotopic mapping dataset: All participants were scanned across 4 MRI sessions on separate nonconsecutive days. 6 task-based runs were acquired each day where participants made active movements (motor runs) as well as 2 runs where participants fixated on a centrally presented black crosshair on a light gray background (fixation runs). In total, each participant had 24 motor and 8 fixation runs. 4 participants (S5–S8) were scanned in an additional session not used here. Each run is about 7 minutes.
8. HCP Unrelated 100 dataset: Two resting scanning sessions per subject. 2 imaging runs per session with each run is about 15 minutes resting scans.

Behavioral performance measures

1. MDTB dataset: Variables recorded: response made, number of correct responses, false alarms, missed responses, response time. Accuracy (% correct) and reaction time (ms) were collected and averaged across tasks per participant.
2. High-res MDTB dataset: Variables recorded: response made, number of correct responses, false alarms, missed responses, response time. Accuracy (% correct) and reaction time (ms) were collected and averaged across tasks per participant.
3. Nishimoto dataset: For 48 out of 103 tasks, task performance was measured using button responses and examined for each participant separately by their median and interquartile range.
4. Individual Brain Charting (IBC) dataset: For ARCHI Standard, HCP Emotion, HCP Gambling, HCP Language, HCP Relational, HCP Social, HCP Working Memory and RSVP Language, active responses were required from the participants. The registry of all behavioral data, such as the qualitative responses to different conditions and corresponding response times, was recorded in log files generated by the stimulus delivery software.
5. WMFS dataset: Variables recorded: Average force (N), Number of taps in 6 seconds. The averaged error rate and its standard deviation was collected for each task condition across subjects.
6. Multi-Demand dataset: Response time and number of correct responses for N-back and Switch task. For stop signal task, go omission, go accuracy, successful stop, unsuccessful stop RT, correct Go RT, and stop signal delay were recorded. Accuracy (% correct) and reaction time (ms) were collected and averaged across tasks per participant (mean ± standard deviation).
7. Somatotopic mapping dataset: Not provided in original paper.
8. HCP Unrelated 100 dataset: Not necessary for resting scans.

Acquisition

Imaging type(s)

EPI, MPRAGE, and GRE field maps

Field strength

Highres-MDTB dataset was acquired at 7T, other datasets were in 3T

Sequence & imaging parameters

1. MDTB dataset: Functional data: A multiband gradient echo-planar imaging sequence (TR = 2,000 ms, TE = 30 ms, flip angle = 62°; voxel size = 2 × 2 × 2 mm, matrix size = 96 × 96, 72 axial slices, FOV = 192 × 192 mm, multiband factor = 3). Structural data: T1-weighted MPRAGE (TR = 2530 ms, TE = 3.26 ms, flip angle = 9°, voxel size = 1 × 1 × 1 mm, matrix size = 256 × 256, 256 axial slices, FOV = 256 × 256 mm).
2. Highres-MDTB dataset: EPI: Gradient echo, multi-band (acceleration factor 3, interleaved) with an in-plane acceleration (factor 3). Imaging parameters were: TR=1s, FOV=19.2cm, PE direction F to H, 60 slices, isotropic 1.5mm3 resolution. For anatomical localization and normalization, a 10-minute high-resolution scan of the whole-brain was acquired (sagittal MP2RAGE, FOV = 24x24x15.6cm3 at isotropic 0.8mm3 voxel size).
3. Nishimoto dataset: Functional data: A multiband gradient echo-planar imaging sequence (TR = 2,000 ms, TE = 30 ms, flip angle = 62°; voxel size = 2 × 2 × 2 mm3, matrix size = 96 × 96, 72 axial slices, FOV = 192 × 192 mm2, multiband factor = 3). Structural data: T1-weighted MPRAGE (TR = 2530 ms, TE = 3.26 ms, flip angle = 9°, voxel size = 1 × 1 × 1 mm3, matrix size = 256 × 256, 256 axial slices, FOV = 256 × 256 mm2).
4. Individual Brain Charting (IBC) dataset: Functional data: A multiband gradient echo-planar imaging sequence (TR = 2,000 ms, TE = 27 ms, flip angle = 74°; voxel size = 1.5 mm isotropic, FOV = 192 × 192 × 140 mm, multiband factor = 3). Structural data: T1-weighted MPRAGE (TR = 2,300 ms, TE = 2.98 ms, flip angle = 9°, voxel size = 1 mm isotropic, matrix size = 256 × 256 × 176 axial slices, FOV = 256 × 256 × 176 mm).
5. WMFS dataset: voxel size = 1 × 1× 1mm, Field-of-view = 25.6 × 25.6 × 25.6 cm, using an echo-planar imaging sequence with TR = 1000 ms, TE = 30 ms, voxel size =2.5×2.5×3 mm, Field-of-view = 20.8×20.8×20.8 cm, 48 slices, P to A phase encoding direction, with multi-band acceleration factor = 3 (interleaved) and in-plane acceleration factor = 2. Gradient echo field maps were acquired to correct for distortions due to B0 inhomogeneities (acquisition parameters: voxel size = 3×3×3 mm, Field-of-view = 24×24×24 cm).
6. Multi-Demand dataset: structural (at least one 3D T1w MPRAGE and one 3D T2w SPACE scan at 0.8-mm isotropic resolution). Whole-brain rest and task fMRI data were acquired using identical multi-band (factor 8) gradient echo EPI sequence parameters of 2-mm isotropic resolution (TR=800 ms, TE=37 ms).
7. Somatotopic mapping dataset: Functional data: (1) For 1.8 mm resolution: A multiband gradient-echo echo-planar pulse sequence (TR = 2,000 ms, TE = 30 ms, flip angle = 80°; voxel size = 2 × 2 × 2 mm, matrix size = 122 × 122 × 87 axial slices, FOV = 220 × 220 mm, multiband factor = 3). (2) For 2.4 mm resolution: TR = 1,000 ms, TE = 33 ms, flip-angle = 64°, matrix 92 × 92 × 65 (FOV = 221 × 221 mm). Structural data: T1-weighted MPRAGE (TR = 2,200 ms, TE = 1.57, 3.39, 5.21, 7.03 ms, flip angle = 7°, voxel size = 1.2 mm, matrix size = 192 × 192 × 176 axial slices).
8. HCP Unrelated 100 dataset: resting-state functional data: A multiband gradient echo-planar imaging sequence (TR = 720 ms, TE = 33.1 ms, flip angle = 52°; voxel size = 2 mm isotropic, matrix size = 104 x 90 (RO x PE), FOV = 208 x 180 mm (RO x PE), multiband factor = 8). Structural data: T1-weighted MPRAGE (TR = 2400 ms, TE = 2.14 ms, TI=1000, flip angle = 8°, voxel size = 0.7 mm isotropic, FOV = 224 × 224 mm).

Area of acquisition

Whole brain scans were used

Diffusion MRI ☐ Used ☒ Not used

Preprocessing

Preprocessing software

1. MDTB dataset: Data preprocessing was carried out using tools from SPM 12, Caret, and SUIT, as well as custom written scripts written in MATLAB 2015b. For all participants, the anatomical image was acquired in the first scanning session and reoriented to align with the Left-Inferior-Posterior (LPI) coordinate frame. Functional data were re-aligned for head motion within each session, and for different head positions.
2. Highres-MDTB dataset: Data preprocessing was carried out using tools from SPM12, optiBET, and SUIT, as well as custom written scripts written in MATLAB 2019b. For all participants, the anatomical image was acquired in the first scanning session and reoriented to align with the Left-Inferior-Posterior (LPI) coordinate frame. Functional data were re-aligned for head motion within each session, and for different head positions across sessions using the 6-parameter rigid body transformation. The mean functional image was then co-registered to the anatomical image, and this transformation was applied to all functional images. No smoothing or anatomical normalization was applied to the functional images.
3. Nishimoto dataset: SPM8 (motion correction) and FreeSurfer 5.3.0 (anatomical registration, cortical surface reconstruction, cortical segmentation, and subcortical segmentation)
4. Individual Brain Charting (IBC) dataset: Source data were preprocessed using PyPreprocess. This library offers a collection of Python tools to facilitate pipeline runs, reporting and quality check (https://github.com/neurospin/pypreprocess). It is built upon the Nipype library43 v0.12.1, that in turn launched various commands used to process neuroimaging data. These commands were taken from the SPM12 software package (Wellcome Department of Imaging Neuroscience, London, UK) v6685, and the FSL library (Analysis Group, FMRIB, Oxford, UK) v5.0.
5. WMFS dataset: Data preprocessing was carried out using tools from SPM 12, SUIT, as well as custom written scripts written in MATLAB 2018b. For all participants, we defined an individual coordinate system for each subject by setting the origin of the anatomical image to the approximate location of the anterior commissure. Functional data were re-aligned for head motion within each session, and for different head positions.
6. Multi-Demand dataset: The HCP pipelines versions 3.27.0 were used (scripts available at: https://github.com/Washington-University/HCPpipelines) for data preprocessing. For each subject, structural images (T1w and T2w) were used for extraction of cortical surfaces and segmentation of subcortical structures. Functional images (rest and task) were mapped from volume to surface space and combined with subcortical data in volume to form the standard CIFTI grayordinates space. Data were smoothed by a 2mm FWHM kernel in the grayordinate space that avoids mixing data across gyral banks for surface data and

avoids mixing across major structure borders for subcortical data.
7. Somatotopic mapping dataset: A custom analysis pipeline for individualized data processing was used, as described in (Bragaet et al.,2019) Briefly, the pipeline combines tools from FreeSurfer, FSL, and AFNI to align data within an individual across runs and sessions to a high-resolution output target (1 mm isotropic) using a single interpolation to minimize spatial blurring.
8. HCP Unrelated 100 dataset: We used the HCP preprocessed data; preprocessing softwares included FSL 5.0.6, FreeSurfer 5.3.0-HCP, and Connectome Workbench v1.1.1.

Normalization	The probabilistic maps for the cerebellum were normalized into SUIT space using the diffeomorphic anatomical registration (DARTEL) algorithm. This algorithm deforms the cerebellum to simultaneously fit the probability maps of cerebellar gray and white matter onto the SUIT atlas template. This non-linear deformation was applied to both the anatomical and functional data. The activation estimates (i.e., the beta weights or resting-state functional connectivity), and residual mean-square images from the first-level GLM were resliced into SUIT space.
Normalization template	For all datasets, the spatially unbiased infratentorial template (SUIT) toolbox (v3.2) in SPM 12 was used to isolate the cerebellum from the rest of the brain and to provide a normalization to a spatially unbiased template of the cerebellum.
Noise and artifact removal	The cerebellar isolation mask was hand corrected to ensure that it did not contain any shared voxels between the superior cerebellum and the directly abutting cerebral cortical regions of the inferior temporal and occipital cortex.
Volume censoring	n/a

Statistical modeling & inference

Model type and settings	First level analysis, (1) task-based fMRI data: a mass-univariate General Linear Model (GLM) was then fitted to the realigned functional data to estimate brain activation per imaging run. Coefficients of the GLM were divided by the root-mean-square error (RMSE) for each voxel, resulting in individual volume-based maps of normalized activity estimates. (2) resting-state functional connectivity: We first concatenated the preprocessed functional data temporally across subjects, sessions, and runs to create a single matrix. Then we used the group ICA implemented in FSL's MELODIC (Jenkinson et al., 2012) with automatic dimensionality estimation, resulting in 1072 group-level components. 69 signal components were identified from the first 300 ICA components as resting-state networks. Lastly, we regressed the 69 group network spatial maps into the subject-and-run-specific cortical time series, resulting in 69 cortical network time courses. The cerebellar rs-FC fingerprints were calculated as Pearson's correlations of the cerebellar voxel time series with each cortical network time course.<br><br>Second level analysis, We used a hierarchical Bayesian framework to learn a functional atlas of the cerebellum from multiple fMRI datasets as described in the manuscript. Variational Inference was applied to deal with the intractability of the model.
Effect(s) tested	*Define precise effect in terms of the task or stimulus conditions instead of psychological concepts and indicate whether ANOVA or factorial designs were used.*

Specify type of analysis: ☒ Whole brain ☐ ROI-based ☐ Both

Statistic type for inference<br>(See Eklund et al. 2016)	Voxel-wise
Correction	Given that we used an uniformed evaluation criterion (DCBC), no correction for the number of voxel-wise or region-wise tests were necessary.

Models & analysis

n/a	Involved in the study
☐	☒ Functional and/or effective connectivity
☒	☐ Graph analysis
☐	☒ Multivariate modeling or predictive analysis

Functional and/or effective connectivity	Resting-state functional connectivity is measured using Pearson correlation of the fMRI time series (static measures) and the model-parameter of a 1-st order autoregressive model of the fMRI time series (dynamic measures).
Multivariate modeling and predictive analysis	We developed a novel hierarchical Bayesian framework to build probabilistic brain parcellations from multiple fMRI dataset (Zhi et al., 2023). From Bayesian modeling perspective, the spatial arrangement model provides group probability prior, and emission models calculate subject-specific data likelihood from each of individual dataset. To make it a probabilistic measure, the parcel assignment for each brain voxel i is designed to be a multinomial random variable over all K possible functional regions. Then these vectors are collected into the K (parcels) × P (voxels) matrix served as central quantity of the framework.<br>The model training was performed under EM algorithm procedures, which globally maximize the objective function. Due to the intractability of the energy-based model, the objective function is the evidence lower bound given the variational inference. When learning starts, the emission models first calculate data likelihood for each subject and pass them to the arrangement model. Then, arrangement model calculate the posterior probability of parcel assignment for every subject. Then these individual parcellations pass back to the emission models to calculate the data likelihood for the next iteration. Then we learn the full model

through multiple EM process until meeting our converge criterion.

To evaluate the performance of the estimated parcellations from trained model, we applied DCBC evaluation (Zhi et al., 2022) throughout. The DCBC method evaluates how well a parcellation corresponds to the functional boundaries in an independent test dataset. A higher DCBC value indicates a better performance of the given parcellation. This evaluation also allows a direct comparison between parcellations in different resolutions and modalities.

As a second evaluation criterion we assessed the ability of a given parcellation to predict functional responses individual held out data, by calculating a prediction error. We first derived the individual parcellations from one half of each dataset, and converted these to winner-take all maps. We then used the data from N-1 subjects of the second half to estimate the mean functional profiles for each region. For each voxel in the Nth subject, we then used the profile of the assigned region as a prediction and calculated the prediction error as one minus the cosine similarity of prediction and data vector. When averaging these results across voxels, we weighted each cosine error by the length of the data vector to ensure that voxels with high signal strength would influence our evaluation more than noisy voxels.
